# Cell-type specific inhibitory plasticity in subicular pyramidal cells

**DOI:** 10.3389/fncel.2024.1368627

**Published:** 2024-04-23

**Authors:** Alix Guinet, Sabine Grosser, Duru Özbay, Joachim Behr, Imre Vida

**Affiliations:** ^1^Institute for Integrative Neuroanatomy, Charité - Universitätsmedizin Berlin, Berlin, Germany; ^2^Institute for Biology, Humboldt - Universität zu Berlin, Berlin, Germany; ^3^Department of Psychiatry, Psychotherapy and Psychosomatic Medicine, Brandenburg Medical School, Neuruppin, Germany; ^4^Department of Psychiatry and Psychotherapy, Charité - Universitätsmedizin Berlin, Berlin, Germany; ^5^Faculty of Health Science Brandenburg, Joint Faculty of the University of Potsdam, Brandenburg University of Technology Cottbus-Senftenberg and Brandenburg Medical School Theodor Fontane, Potsdam, Germany

**Keywords:** subiculum, pyramidal cells, GABAergic inhibition, synaptic plasticity, hippocampus, rat

## Abstract

The balance between excitation and inhibition is essential to the proper function of cortical circuits. To maintain this balance during dynamic network activity, modulation of the strength of inhibitory synapses is a central requirement. In this study, we aimed to characterize perisomatic inhibition and its plasticity onto pyramidal cells (PCs) in the subiculum, the main output region of the hippocampus. We performed whole-cell patch-clamp recordings from the two main functional PC types, burst (BS) and regular spiking (RS) neurons in acute rat hippocampal slices and applied two different extracellular high-frequency stimulation paradigms: non-associative (presynaptic stimulation only) and associative stimulation (concurrent pre-and postsynaptic stimulation) to induce plasticity. Our results revealed cell type-specific differences in the expression of inhibitory plasticity depending on the induction paradigm: While associative stimulation caused robust inhibitory plasticity in both cell types, non-associative stimulation produced long-term potentiation in RS, but not in BS PCs. Analysis of paired-pulse ratio, variance of IPSPs, and postsynaptic Ca2+ buffering indicated a dominant postsynaptic calcium-dependent signaling and expression of inhibitory plasticity in both PC types. This divergence in inhibitory plasticity complements a stronger inhibition and a higher intrinsic excitability in RS as compared to BS neurons, suggesting differential involvement of the two PC types during network activation and information processing in the subiculum.

## 1 Introduction

The subiculum, located between the hippocampus proper and the entorhinal cortex, acts as an important output station, critically involved in hippocampal learning and memory consolidation (Deadwyler and Hampson, 2004; O’Mara, 2006; Wozny et al., 2008a,b; Behr et al., 2009; Fidzinski et al., 2012), as well as in pathological processes, such as epilepsy (Miles et al., 2012; Grosser et al., 2015; Buchin et al., 2016; Grosser et al., 2020). It comprises two functional types of excitatory principal neurons: BS and RS PCs (Jung et al., 2001; Menendez de la Prida et al., 2003). Upon depolarization, BS neurons show an initial burst of high-frequency spiking action potentials (APs). The initial burst can be followed by further bursts or a sequence of single APs (Staff et al., 2000). In contrast, RS neurons fire a train of single action potentials. The two types, however, not only differ in their physiology, but also in their local connectivity (Böhm et al., 2015) and long-range projection (Kim and Spruston, 2012; Cembrowski et al., 2018), therefore they may be involved in different information processing streams channeled through the subiculum.

Similar to other brain structures, PCs in subicular circuit also interact with a highly heterogeneous group of GABAergic inhibitory interneurons (IN) (Freund and Buzsáki, 1996; Vida et al., 1998). Interneurons can be classified based on their postsynaptic targets, including those that innervate the perisomatic or dendritic domains of PCs, and those that preferentially target other interneurons. Of particular interest are perisomatic inhibitory parvalbumin-positive (PV), fast-spiking basket cells, which comprise 30–40% of interneurons in different cortical areas (Booker and Vida, 2018). Given the central localization of their synapses on the target neurons, they have high functional impact at the cellular (Pouille and Scanziani, 2001; Strüber et al., 2022) and network levels (Bartos et al., 2007).

Through the interaction of PC and interneurons a dynamic balance of excitation and inhibition ensues in the local network, supported by feedforward and feedback circuit motifs. The dynamic balance is presumed to be important for the emergence of network activity patterns and for the processing of information (Vogels et al., 2005). The long-term maintenance of this balance involves synaptic plasticity. Long-term potentiation (LTP) at excitatory synapses has been well characterized in the subiculum (Wozny et al., 2008a,b; Behr et al., 2009; Grosser et al., 2020), in contrast, little is known about inhibitory synaptic plasticity in this region. In fact, since the seminal finding of LTP in the hippocampal CA1 by Bliss and Lømo (1973), plasticity of excitatory synapses has been intensely investigated in many different cortical areas, whereas the existence of inhibitory plasticity has been recognized substantially later in the developing visual (Komatsu and Iwakiri, 1993) and cerebellar cortex (Kano et al., 1992), but since then, a plethora of diverse forms of inhibitory plasticity have been identified in various brain regions (Castillo et al., 2011; Maffei, 2011), including the hippocampal CA1 (Patenaude et al., 2003; Jappy et al., 2016). Whether inhibitory long-term plasticity (iLTP) can be induced in the subiculum, remains an open question. In this study, we aimed to investigate perisomatic inhibitory plasticity in subicular BS and RS PCs by combining whole-cell patch-clamp recordings and different patterns of focal extracellular stimulation.

## 2 Materials and methods

All experiments were performed in accordance with European Council Directive 86/609/EEC, the German Animal Welfare Act, and local authority guidelines (Berlin, T-CH 0020/40).

### 2.1 Preparation of acute horizontal brain slices

Acute hippocampal slices were obtained from wild-type or genetically modified Wistar rats (p20-30), expressing the fluorescent Venus/YFP protein under the Vesicular GABA transporter (vGAT) promoter (Uematsu et al., 2008). Female and male animals were equally used for experiments. Animals were decapitated after deep isoflurane anesthesia (3%), brains were quickly removed and transferred into ice-cold, carbogenated (95% O_2_, 5% CO_2_), sucrose-based artificial cerebrospinal fluid (sACSF) containing (in mM): 87 NaCl, 2.5 KCl, 25 NaHCO_3_, 1.25 NaH_2_PO_4_, 25 Glucose, 75 Sucrose, 1 Na_2_-Pyruvate, 1 Na_2_-Ascorbate, 7 MgCl_2_, 0.5 CaCl_2_; pH 7.4, 340 to 350 mOsm/L. Horizontal brain slices of 300 μm thickness were cut using a vibratome (VT1200S, Leica, Germany) and collected in sACSF in a submerged holding chamber and were allowed to recover at 32–34°C for 30 min. Subsequently, slices were stored at room temperature in sACSF until recording. Chemicals and reagents were purchased from the following companies: Merck (Germany), Roth (Germany), Sigma-Aldrich (United States), if not stated otherwise.

### 2.2 Electrophysiology

For whole-cell patch-clamp recordings, slices were transferred to a submerged recording chamber (Grosser et al., 2021). The chamber was continuously superfused (5 mL/min) with carbogenated ACSF containing (in mM): 125 NaCl, 2.5 KCl, 25 NaHCO3, 1.25 NaH2 PO4, 25 glucose, 1 MgCl2, 2 CaCl2, 1 Na-pyruvate, 1 ascorbic acid, 320 mOsm/L and maintained at 32–34°C. A horizontal puller (P-97, Sutter Instruments, CA, United States) was used to draw custom patch pipettes from borosilicate glass capillaries (Hilgenberg, Germany) with an input resistance of 5–6 MΩ. Two intracellular solutions with different EGTA concentrations were used in this study. One intracellular solution with a low EGTA concentration contained (in mM): 135 K-gluconate, 10 KCl, 2 MgCl2, 0.1 EGTA, 10 HEPES, 2 Na2-ATP, 0.3 Na2-GTP, 1 Na2-creatinine, 0.1% biotinylated lysine. The other intracellular solution with a high EGTA concentration contained (in mM): 130 K-gluconate, 10 KCl, 2 MgCl2, 10 EGTA, 10 HEPES, 2 Na2-ATP, 0.3 Na2-GTP, 1 Na2-creatinine, 0.1% biotinylated lysine.

Neurons were selected and targeted for whole-cell recordings under visual guidance using an upright microscope (BX51WI, Olympus, Japan) equipped with an infrared light source, oblique illumination, and a digital camera (RetigaTM ELECTRO, Teledyne QImaging, United Kingdom). Electrophysiological data was recorded in current-clamp mode using an amplifier (Axopatch 200B, Molecular Devices, CA, United States). Signals were filtered at 10 kHz using the built in low-pass filter of the amplifier and digitized at 20 kHz (PCIe-6321 National Instruments, TX, United States). Data was collected using the open-source WinWCP software package (courtesy of Dr. J. Dempster, University of Strathclyde, Glasgow, https://spider.science.strath.ac.uk) and analyzed offline using Stimfit.^1^ To assess intrinsic properties and discharge patterns, a family of current steps ranging from-250 pA to +250 pA (50 pA steps, 500 ms duration) was applied. Passive membrane properties were derived from voltage responses to a-50 pA current pulse. Input resistance was calculated taking the difference between baseline and steady state of the response according to Ohm’s Law. Membrane time-constant was estimated by fitting the mono-exponential curve after pulse application until repolarization of the membrane potential. Resting membrane potential (RMP) was calculated by averaging 50 ms of initial baseline. Active membrane properties were analyzed from single APs elicited at rheobase. Voltage threshold was measured at the point where the first derivative of the AP reached 20 mV/ms. AP amplitude was measured from threshold. AP maximal rise and decay rates were taken as the maxima of the first derivative of the ascending and descending phases of the AP. The rheobase was estimated as the first depolarizing current step to elicit an AP in the recorded neuron. Afterhyperpolarization (AHP) was measured from threshold to the negative peaks: (1) immediately after an AP (fast AHP), and (2) the more pronounced delayed negative deflection (medium AHP). Slow AHP was measured directly after trains evoked by depolarizing current pulses (250 pA, 500 ms) and calculated from preceding baseline to the maximal negative deflection. The maximum firing frequency was determined during current injection of 250 pA. Finally, sag potential was measured during a hyperpolarizing response to a pulse of-250 pA, as the difference between the negative peak and steady state.

**Figure 1:**
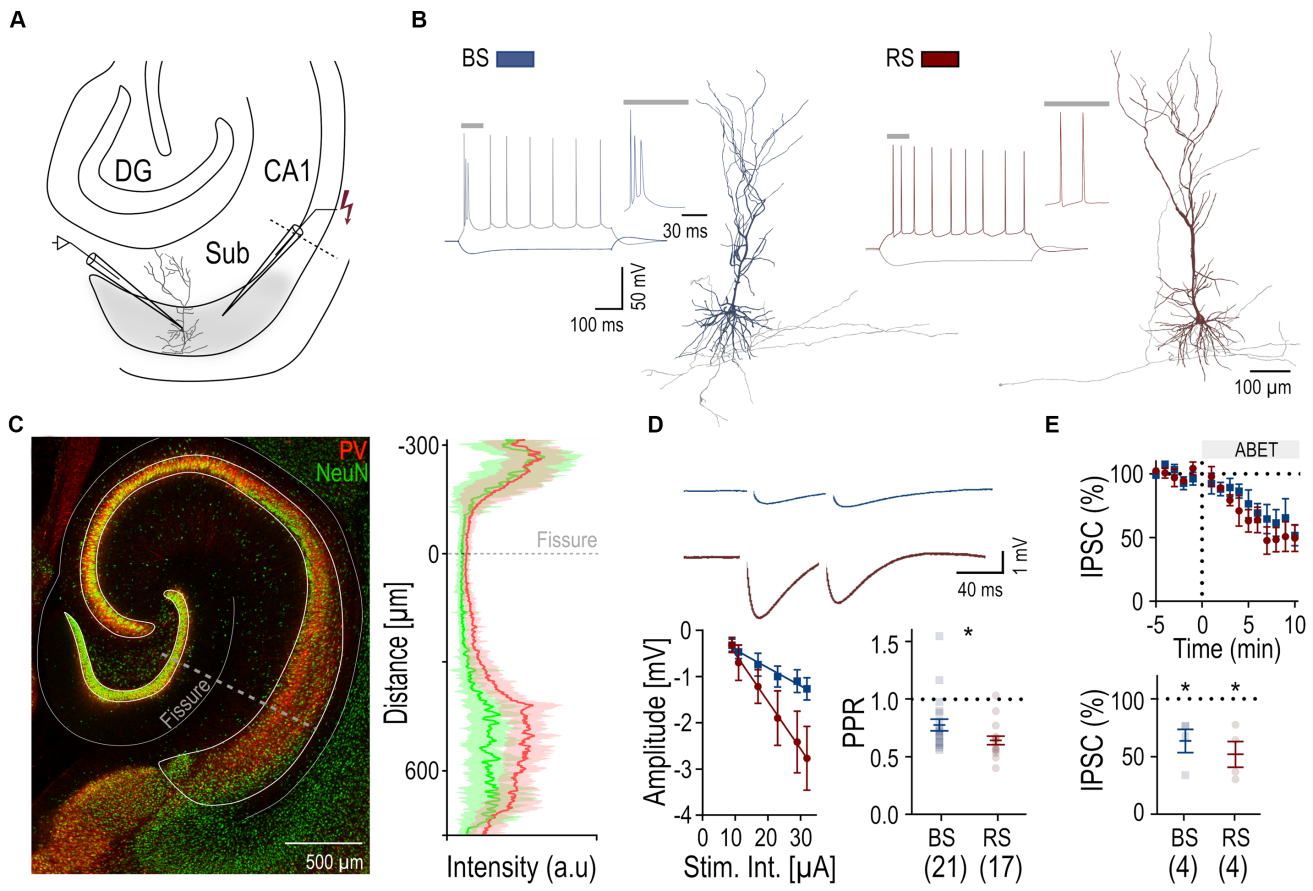
Perisomatic inhibition in BS and RS PCs. **(A)** Schematic drawing illustrating the placement of the recording (left) and stimulation (right) electrode in the subiculum (Sub) of a hippocampal slice. **(B)** Firing patterns of a BS (blue) and a RS (red) neuron in response to a de-and hyperpolarizing current pulses (+ 250pA; − 250pA). Insets show initial firing. Sample reconstructions of corresponding neurons: BS (blue) and RS (red). **(C)** Left: Merged confocal image of immunostaining for PV (red) and NeuN (green) in a hippocampal slice. Right: Superimposed intensity profile for PV (red) and NeuN (green), mean values are displayed as solid lines, SD is indicated by transparent outlines. **(D)** Top: Monosynaptic IPSPs recorded from a BS (*n* = 21) and a RS neuron (*n* = 17), evoked by paired extracellular stimulation under baseline conditions. Bottom left: IPSP amplitudes plotted as a function of the stimulus intensities for the two PC types, BS (*n* = 8) and RS neurons (*n* = 6). Bottom right: Summary box charts of PPR of IPSP amplitudes for the two PC types under baseline conditions. **(E)** Top: Time Course plot of the mean IPSC amplitude in BS and RS neurons testing pharmacological sensitivity to M2R agonist (ABET). Time 0 corresponds to the time of drug application. Bottom: Summary chart of the normalized mean IPSCs in BS and RS neurons between 5 and 10 min after drug application. Significance levels: * *p* < 0.05, DG, Dentate Gyrus; CA1, Cornu Ammonis 1.

To evoke synaptic responses, a monopolar stimulation electrode (a pipette filled with 2 M NaCl) was positioned in the upper pyramidal cell layer at a distance of approximate 100–150 μm from the recorded cell (Figure 1A). To isolate monosynaptic inhibitory postsynaptic potentials (IPSPs) or currents (IPSCs), ionotropic glutamate receptor antagonists 6,7-Dinitroquinoxaline-2,3-dione (DNQX, 10 μM, Abcam) and D-2-Amino-5-phosphonopentanoic acid (D-APV, 50 μM, Abcam or Tocris) were added to the ACSF. The stimulus intensity was set slightly above threshold to reliably elicit synaptic responses.

For the induction of inhibitory plasticity, two different stimulation protocols were used in this study: (1) a non-associative stimulation protocol consisting of high frequency presynaptic extracellular stimulation (HFS, frequency: 100 Hz, duration: 1 s, repeated 4 times at 0.1 Hz), and (2) an associative stimulation protocol, consisting of the presynaptic HFS combined with postsynaptic depolarizing pulses (4 pA, 1 s each). Protocols were applied after a stable 5 min baseline of IPSP amplitudes at RMP and the evoked responses were collected subsequently for at least 30 min. To assess the expression of plasticity, averaged IPSP amplitudes, collected between 25 and 30 min after HFS, were normalized to the mean amplitude during the 5 min baseline period preceding the HFS. For time course plots, the IPSP amplitudes were binned into 1 min intervals. Paired stimuli with 50 ms interpulse interval were applied every 10 s. Paired-pulse ratio (PPR) was calculated by dividing the amplitude of the second IPSP by the amplitude of the first IPSP (IPSP_2_/IPSP_1_) (Zucker and Regehr, 2002). The coefficient of variation (CV) of IPSP amplitudes (1/CV^2^ = mean^2^/SD^2^) (van Huijstee and Kessels, 2020) was analyzed and compared between the 5 min baseline and 25–30 min after the HFS.

To examine the currents underlying the synaptic responses, IPSCs were recorded in voltage-clamp mode at a holding potential of – 50 pA in a subset of PCs. Peak amplitude and temporal parameters, as onset latency from stimulus time, 20–80% rise time, and decay time constants (monoexponential fit) were derived from averaged IPSCs (30 traces). To test presynaptic inhibition mediated by muscarinic type 2 receptors (M2R), characteristic for PV IN mediated inhibition (Booker et al., 2017), the agonist arecaidine but-2-ynyl ester tosylate (ABET, 15 μM) was applied to the bath. Drug effect was assessed as the change in the mean IPSC amplitudes between 5 and 10 min after application compared to 5 min of the preceding control period.

### 2.3 Histological processing and immunohistochemistry

Cells were filled with biocytin (Invitrogen) whilst being recorded and subsequently fixed overnight at 4°C in a solution containing 4% paraformaldehyde in 0.1 mM phosphate buffer (PB) and stored in 0.1 mM PB with 0.05% NaN_3_. For histological processing, slices were first washed in 0.1 mM PB and then in 0.1 mM phosphate buffered saline (PBS), permeabilized with Triton X-100 (1%) in PBS and the neurons were visualized by incubation with a streptavidin conjugated fluorochrome (Alexa Fluor 647, Invitrogen, dilution 1:1000) (Grosser et al., 2021).

A subset of slices was immunolabeled for PV and NeuN: After rinsing, a blocking step was performed in 10% normal goat serum (Gibco), 1% Triton X-100 (Serva) and 0.05% NaN_3_ in 0.1 mM PBS. The slices were incubated with a primary antibody against PV (polyclonal guinea pig, 1:1000, Synaptic Systems) and NeuN (polyclonal rabbit, 1:1000, Millipore) for 2 days. Following multiple washing steps in PBS, fluorescent conjugated secondary antibodies were applied for 24 h (anti-guinea pig, 1:500, Invitrogen and anti-rabbit, 1:100, Invitrogen). The slices were mounted in an aqueous mounting medium (Fluoromount-G, Invitrogen) using 300 μm metal spacer to prevent slice shrinkage (Bolduan et al., 2020).

### 2.4 Confocal microscopy

Cells were imaged on a confocal laser scanning microscope (FV1000, Olympus, Japan). Low magnification overviews (4x objective) were used to determine the location of recorded neurons within the subiculum and to obtain intensity profiles of immunolabeled slices. For the latter analysis, labeling intensities for PV and NeuN were measured by generating 5 line profiles per slice (in 4 slices of 3 animals), crossing all layers of the subiculum and extending into the DG. Intensities were averaged and shown in arbitrary units.

Higher magnification image stacks (30x) were used to confirm the identity of PCs (triangular or ellipsoid soma, with multiple basal dendrites arising from the base of the soma, and one, occasionally two, apical dendrite with a distal tuft emerging from the apex of the soma; dendrites densely covered by spines). Stacks were stiched using the ImageJ software^2^ and selected neurons were fully reconstructed for illustration purposes in neuTube.^3^

### 2.5 Statistical analysis

Data is presented as mean ± standard error of the mean (SEM) unless indicated otherwise. Intrinsic physiological and IPSC parameters of BS and RS neurons were tested for normality and compared using either an unpaired parametric Student’s t-test, with Welch correction for unequal variances, if normally distributed, or using a nonparametric Mann–Whitney U test in the case of not normally distributed data or sample sizes with less than 10 recordings. The synaptic input/output functions and pharmacological effects (ABET) were compared using two-way ANOVA. Changes in IPSP amplitudes were tested using Wilcoxon’s test. Group data on plasticity in RS and BS neurons were compared using Mann–Whitney U test. Changes in PPRs and 1/CV2 were evaluated using Wilcoxon’s test. All statistical analysis were performed in GraphPad Prism.^4^

## 3 Results

### 3.1 Membrane properties and inhibitory input in BS and RS PCs

Neuronal networks of the subiculum comprise two functional types of PCs: BS and RS cells, defined by their distinct firing (Menendez de la Prida et al., 2003; Graves et al., 2012; Kim and Spruston, 2012). Indeed, BS and RS neurons recorded from acute rat slices (Figure 1A) could be distinguished based on their discharge pattern in response to suprathreshold depolarizing current pulses: BS neurons (*n* = 21) exhibited an initial burst of APs riding on a depolarizing envelop, followed by further bursts or single APs. In contrast, RS neurons (*n* = 17) fired a train of single APs with moderate accommodation (Figure 1B). Analysis of the intrinsic physiology revealed that BS and RS neurons showed further divergence in their passive and active properties (Table 1): BS neurons had an almost 50% lower input resistance and markedly higher capacitance than RS neurons. Consistent with their reduced input resistance, BS neurons also showed a significantly higher rheobase as well as a lower maximal discharge frequency. Additionally, BS neurons displayed differences in the AP waveform and their afterpotentials, with a significantly slower AP rise rate, a reduced rise and decay ratio, almost no fast AHP and a smaller medium AHP. Finally, BS PCs showed a less pronounced sag potential than RS neurons in response to hyperpolarizing pulses (Table 1).

**Table 1:** Passive and active physiological properties of BS and RS PCs in the subiculum.

	BS (21)	RS (17)	*p*-value	
Passive properties				
Resting membrane potential [mV]	−62.52 ± 0.65	−62.40 ± 0.93	0.92	n.s.
Input resistance [MΩ]	77.44 ± 6.02	159.70 ± 14.88	<0.0001	****
Membrane time constant [ms]	20.06 ± 2.21	21.75 ± 2.21	0.6	n.s.
Membrane capacitance [pF]	274.9 ± 30.04	150.7 ± 17.03	0.001	**
Active properties				
Sag amplitude [mV]	−4.75 ± 0.33	−8.07 ± 0.66	<0.001	***
AP amplitude [mV]^a^	80.14 ± 2.36	74.38 ± 2.52	0.07	n.s.
AP rise time [ms]	0.21 ± 0.01	0.23 ± 0.01	0.15	n.s.
AP rise rate [mV/ms]	307 ± 24.36	240.9 ± 20.64	0.046	*
AP decay rate [mV/ms]	79.46 ± 5.35	72.75 ± 3.42	0.3	n.s.
Rise/decay ratio	3.82 ± 0.12	3.26 ± 0.18	0.02	*
AP half-width [ms]	1.08 ± 0.06	1.12 ± 0.05	0.65	n.s.
Fast AHP amplitude [mV]^a^	0.61 ± 0.71	−7.41 ± 1.19	<0.0001	****
Medium AHP amplitude [mv]^a^	−9.43 ± 0.56	−11.64 ± 0.30	0.004	**
Slow AHP amplitude [mV]^b^	−4.17 ± 0.27	−4.21 ± 0.47	0.94	n.s.
Max discharge frequency [Hz]	18.62 ± 2.0	24.03 ± 1.78	0.04	*
Rheobase [mV]	183.3 ± 26.58	117.6 ± 10.45	0.04	*

a Measured from threshold.

b Measured from baseline.

* *p* < 0.05, ** *p* < 0.01, *** *p* < 0.001, **** *p* < 0.0001.

Recorded neurons were intracellularly filled and subsequently visualized to confirm their identity as PCs (Figure 1B) and determine their location within the subiculum. Immunohistochemical staining against PV, a marker of fast-spiking perisomatic inhibitory interneurons, and NeuN, a neuronal marker, was performed in a subset of slices (Figure 1C). It revealed strong variations in PV and NeuN immunolabeling intensity across the layers, being high in the cell body layers of all hippocampal areas, where somata of principal cells are localized, and low in dendritic layers (Figure 1C). Within the subiculum we further noted a more subtle difference within the cell body layer along the superficial—deep axis: the labeling was high in the superficial part and declined towards the depth, suggesting changing densities of PV+ axon collaterals along the vertical axis (Figure 1C).

In order to analyze the perisomatic inhibitory input of the recorded PCs, we applied extracellular stimulation using a monopolar electrode placed in the cell body layer adjacent (100–150 μM distance) to the recorded neurons in the presence of fast ionotropic glutamate receptor blockers (APV 50 μM and DNQX 10 μM). Pharmacologically isolated, monosynaptic IPSPs could be reliably elicited in both PC types (Figure 1). Comparison of IPSPs as a function of stimulus intensity showed larger amplitudes in RS (*n* = 6) than in BS neurons (*n* = 8) (two-way ANOVA, factor cell type: *F* (1,72) = 12.82, *p* = 0.0006; factor intensity: *F* (5,72) = 7.08, *p* < 0.0001; with no interaction: *F* (5,72), *p* = 0.22; Figure 1D). In response to the paired stimuli, amplitudes of the second IPSPs were lower in both PC types (Figure 1D). However, paired pulse depression was less pronounced in BS neurons (PPR: 0.78 ± 0.05, *n* = 21) than in RS neurons (PPR: 0.64 ± 0.04, *n* = 17, *p* = 0.01).

IPSCs underlying the evoked synaptic responses were analyzed in voltage-clamp recordings from a subset of BS (*n* = 8) and RS neurons (*n* = 9) (Supplementary Figure S1). The peak amplitude: RS: 52.5 ± 8.7; *p* = 0.5 of the IPSCs was comparable in the two cell types (BS: 42.4 pA ± 8.7; RS cells: 45.1 pA ± 5.4; *p* = 0.8), suggesting that the difference in the IPSP amplitudes was mainly due to divergence in the input resistance of PCs. Furthermore, the kinetic properties of the IPSCs were also similar in the two cell types. Neither the onset latency: RS: 2.59 ± 0.3; 08 RS: 2.64 ms ± 0.3; *p* = 0.7), nor the rise time: 1.7 ± 0.3; *p* = 0.6; RS: 1.77 ms ± 0.3; *p* = 0.5) or the decay: RS: 13.8 ± 1.9, *p* = 0.7 RS: 12.39 ms ± 1.4; *p* = 0.4) showed statistically significant difference between BS and RS neurons.

Finally, to test presynaptic modulation of the synaptic responses by M2R, a characteristic of PV-mediated perisomatic inhibition (Chiang et al., 2010; Booker et al., 2017), evoked IPSCs were recorded and the M2R agonist ABET (15 μM) was bath applied. Both cell types showed a comparable reduction in IPSC peak amplitudes (BS: 37% lower amplitude, *n* = 4; RS: 48% lower amplitude, *n* = 4; Figure 1E). Two-way ANOVA indicated a significant pharmacological effect (*F* (1,12) = 5,16, *p* = 0.04), but no difference between PC types (*F* (1,12) = 0.15, *p* = 0.71) and no interaction between the treatment and the cell types (*F* (1,12) = 0.05, *p* = 0.83).

In summary, our electrophysiological analysis revealed differences between BS and RS neurons in their intrinsic, as well as functional synaptic properties, including the amplitude of IPSPs and their short-term plasticity. However, no differences were found between the amplitudes and kinetics of underlying IPSCs and their pharmacological sensitivity to an M2R agonist, suggesting that the evoked inhibitory synaptic response were predominantly meditated by perisomatic PV interneurons in both PC types.

### 3.2 Non-associative stimulation induces GABAergic iLTP in RS, but not in BS PCs

**Figure 2:**
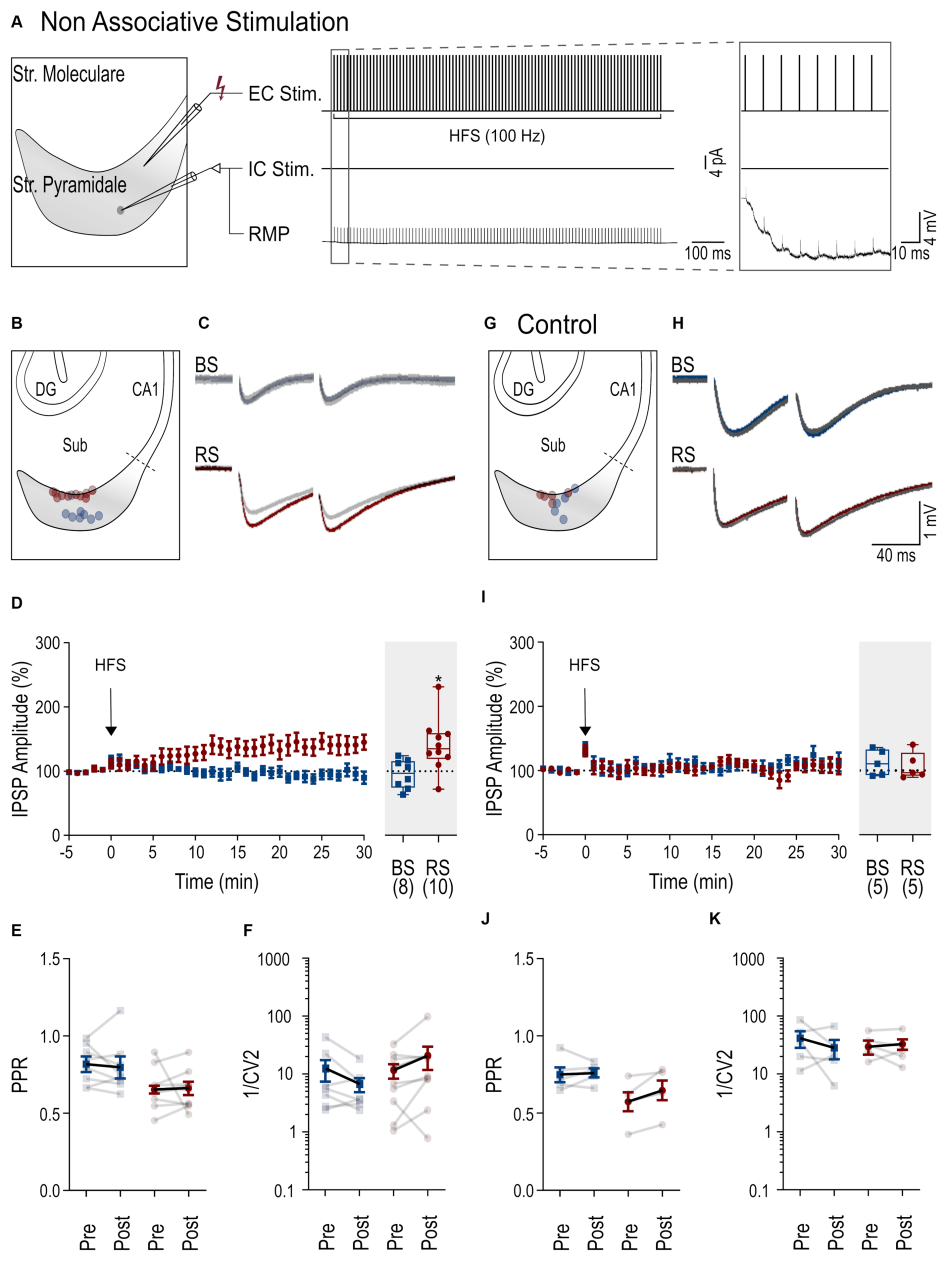
Cell-dependent iLTP following non-associative stimulation paradigm. **(A)** Scheme of the non-associative induction paradigm: extracellular HFS applied to presynaptic axons (top trace, EC Stim.) without a postsynaptic current pulse to the neuron (middle trace, IC. Stim) and recorded membrane potential from the neuron (bottom trace, RMP). Inset to the right shows the initial part of the HFS at higher temporal resolution. **(B)** Schematic of the subiculum (Sub) illustrating the locations of the recorded BS (blue) and RS neurons (red). **(C)** Representative IPSPs from a BS and a RS neuron evoked by paired pulse stimuli during baseline period (in gray, average over 5 min) and after HFS (color coded, average between 25 and 30 min after HFS). **(D)** Left: Time course plot of the mean IPSP amplitudes in BS and RS neurons during the experiments. Time 0 corresponds to the start of the HFS. Data was normalized to baseline and binned to 1 min intervals. Inset on the right: Summary chart of the normalized mean IPSP amplitudes in BS and RS neurons between 25 and 30 min after HFS. **(E)** Plot of the PPR of IPSP amplitudes before (pre-HFS) and after the HFS (post-HFS) in BS and RS neurons. Values from the same neurons are connected by lines; mean ± SEM values are in bold. **(F)** Plot of the 1/CV^2^ values from BS and RS neurons before (pre-HFS) and after the HFS (post-HFS). Values from the same neurons are connected by lines; mean ± SEM values are in bold. **(G–K)** Illustration of corresponding experimental results using an intracellular solution with a high concentration of EGTA (10 mM) as in panels **(B–F)**. Significance levels: * *p* < 0.05, CA1, Cornu Ammonis 1; DG, Dentate Gyrus; Sub, Subiculum.

To investigate inhibitory plasticity, next, a HFS (Wozny et al., 2008b) was applied via the extracellular electrode to the presynaptic inhibitory axons (non-associative stimulation paradigm, Figure 2A). Subsequent identification and localization of the recorded PCs showed a spatial segregation of the recorded neurons with BS PCs in the deep and RS PCs in the superficial cell body layers of the subiculum (Figure 2B) as reported earlier (Cembrowski et al., 2018). In BS PCs, no change of the IPSP amplitude was observed following the HFS protocol (25–30 min, 95.5 ± 7.9% of baseline amplitude, *n* = 8, *p* = 0.64, Figures 2C,D). In contrast, IPSP amplitudes in RS neurons gradually increased and reached 140.8 ± 13.1% of baseline level (*n* = 10, *p* = 0.014, Figure 2D). To determine whether iLTP was expressed pre-or postsynaptically, we compared the PPR of IPSP amplitudes before and after the HFS in RS PCs. We found no change in the PPR values between baseline (0.65 ± 0.04) and 25–30 min after HFS (0.66 ± 0.04, *p* = 0.19, Figure 2E), indicating a postsynaptic expression. Likewise, the analysis of the inverse square of the coefficient of variation (1/CV^2^) of IPSP amplitudes (van Huijstee and Kessels, 2020) did not show any change in RS neurons (*p* = 0.07, Figure 2F), further supporting the notion of postsynaptic expression. In BS neurons, given the lack of plasticity, neither PPR no 1/CV^2^ changed.

To study the postsynaptic mechanism of iLTP in more detail, a higher concentration of the calcium chelator EGTA (10 mM) was used for the intracellular solution. Under this condition, no increase in the IPSP amplitude was observed in BS (112.3 ± 9% of baseline amplitude, *n* = 5, *p* = 0.31, Figures 2H,I) or RS PCs (107.4 ± 9.4% of baseline amplitude, *n* = 5, *p* = 0.81), indicating that an interference with intracellular calcium signaling blocks iLTP. Similarly, PPR and 1/CV^2^ values remained unchanged in both cell types (Figures 2J,K). In summary, our results show that a non-associative stimulation paradigm induces cell type-specific, calcium-dependent iLTP at perisomatic inhibitory synapses onto subicular RS, but not onto BC PCs.

### 3.3 Associative stimulation induces GABAergic iLTP in both BS and RS PCs

**Figure 3:**
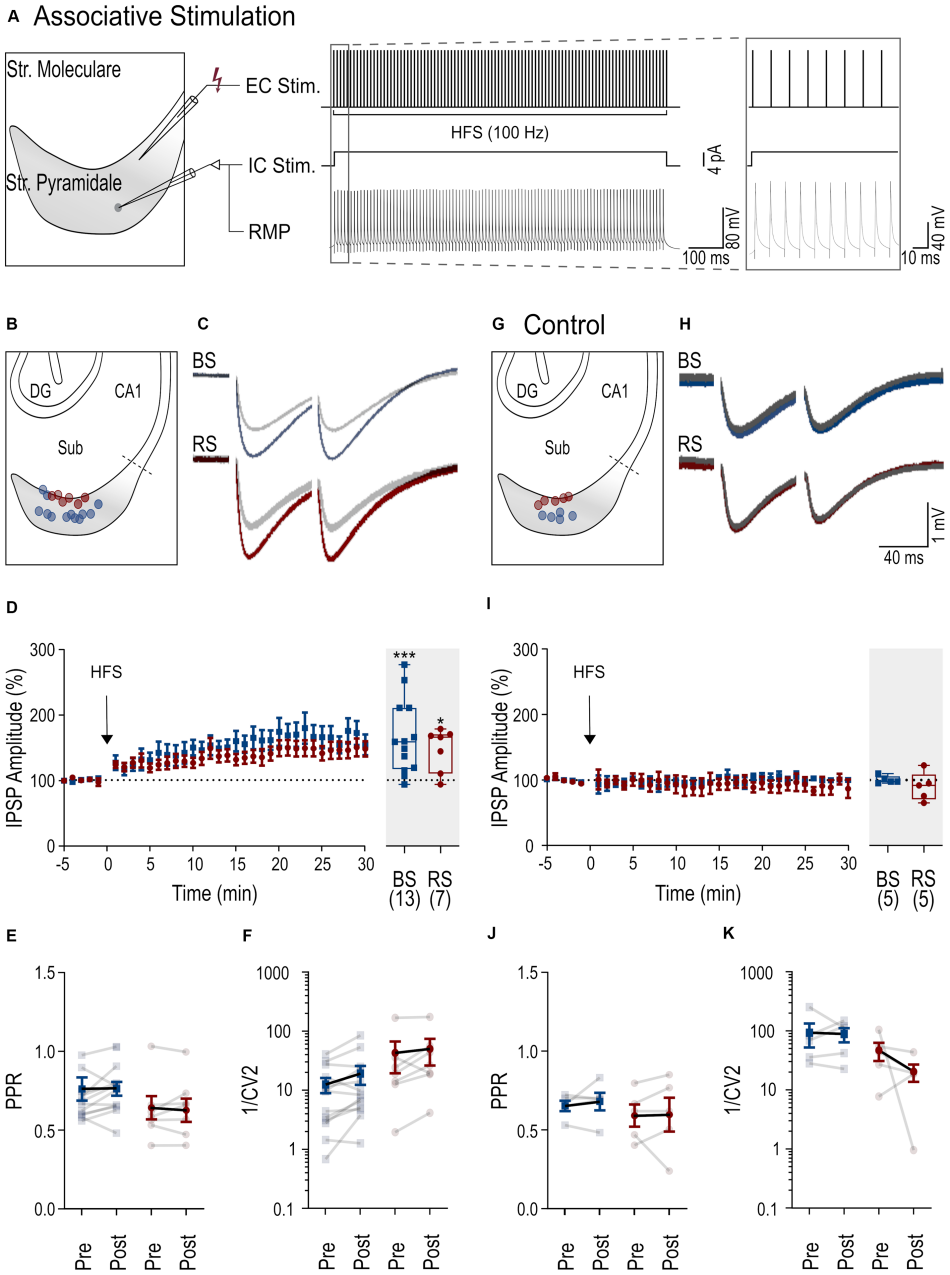
iLTP following associative stimulation in RS and BS PCs. **(A)** Scheme of the associative induction paradigm: extracellular HFS applied to presynaptic axons (top trace, EC Stim.) combined with a postsynaptic depolarizing current pulse (4 pA) to the neuron (middle trace, IC. Stim) and the recorded membrane potential from the neuron (bottom trace, RMP). Inset to the right shows the initial part of the HFS at higher temporal resolution. **(B)** Schematic of the subiculum (Sub) illustrating the locations of the recorded BS (blue) and RS neurons (red). **(C)** Representative IPSPs from a BS and a RS neuron evoked by paired pulse stimuli during baseline period (in gray, average over 5 min) and after HFS (color coded, average between 25 and 30 min after HFS). **(D)** Left: Time course plot of the mean IPSP amplitudes in BS and RS neurons during the experiments. Time 0 corresponds to the start of the HFS. Data was normalized to baseline and binned to 1 min intervals. Inset on the right: Summary chart of the normalized mean IPSP amplitudes in BS and RS neurons between 25 and 30 min after HFS. **(E)** Plot of the PPR of IPSP amplitudes before (pre-HFS) and after the HFS (post-HFS) in BS and RS neurons. Values from the same neurons are connected by lines; mean ± SEM values are in bold. **(F)** Plot of the 1/CV^2^ values from BS and RS neurons before (pre-HFS) and after the HFS (post-HFS). Values from the same neurons are connected by lines; mean ± SEM values are in bold. **(G–K)** Illustration of corresponding experimental results using an intracellular solution with a high concentration of EGTA (10 mM) as in panels **(B–F)**. Significance levels: * *p* < 0.05, *** *p* < 0.001, CA1, Cornu Ammonis 1; DG, Dentate Gyrus; Sub, Subiculum.

Inhibitory synaptic plasticity has been found to exhibit substantial heterogeneity (Patenaude et al., 2003; Jappy et al., 2016) depending on the stimulation paradigm, induction site, and target cell type. Therefore, next, we applied an associative stimulation paradigm, combining presynaptic HFS with postsynaptic depolarization (Figure 3A) to test the induction of iLTP in subicular PCs. In response to this induction protocol, both PC types displayed a robust increase of IPSP amplitudes (BS neurons: 165.4% ± 15.7 of baseline amplitude, n = 13, *p* = 0.001; RS neurons: 147 ± 12.4% of baseline amplitude, n = 7, *p* = 0.031, Figures 3C,D). There was no change in the PPR (baseline in BS: 0.76 ± 0.07, after HFS: 0.76 ± 0.04, *p* = 0.38; baseline in RS: 0.63 ± 0.07, after HFS: 0.64 ± 0.07, *p* = 0.47, Figure 3E) or the 1/CV^2^ (BS: p = 0.07; RS: *p* = 0.16, Figure 3F), indicating a postsynaptic site of expression in both cell types in this induction paradigm.

To test for postsynaptic calcium-dependence of iLTP in the associative induction paradigm, high concentration of EGTA (10 mM) was used in the intracellular solution in another set of experiments (Figures 3H,I). Under these conditions, the associative stimulation did not induce potentiation of the IPSP amplitudes in either of the two PC types (BS: 100.7 ± 2.6% of baseline amplitude, *n* = 5, *p* = 0.81; in RS: 90.1% ± 9.7 of baseline amplitude, *n* = 5, *p* = 0.31). No significant changes in PPRs or in 1/CV^2^ were observed in BS and RS neurons (Figures 3J,K). In summary, in contrast to non-associative stimulation, associative stimulation readily induces calcium-dependent iLTP at perisomatic inhibitory synapses in both BS and RS neurons.

## 4 Discussion

In the present study, we investigated perisomatic inhibitory plasticity in the two functional PC types of the subiculum: RS and BS neurons. We found that, while iLTP is expressed in both types, the induction shows cell type-specific differences, with RS, but not BS pyramidal cells, readily showing plasticity in response to non-associative purely presynaptic stimulation. In both PC types iLTP was calcium dependent and showed hallmarks of a postsynaptic expression.

### 4.1 Intrinsic properties indicate higher excitability in RS than in BS PCs

Pyramidal cells of the subiculum have been classified as RS and BS PCs based on their distinct discharge pattern (Taube, 1993; Behr et al., 1996; Staff et al., 2000; Menendez de la Prida et al., 2003; Wozny et al., 2008b; Behr et al., 2009; Graves et al., 2012; Kim and Spruston, 2012). In the present study, while the two types showed largely similar intrinsic electrophysiological properties, consistent with them being pyramidal cells (Taube, 1993; Staff et al., 2000; Wozny et al., 2008b), we also found a number of quantitative differences in passive and active membrane properties beyond their distinct discharge pattern (Menendez de la Prida et al., 2003; Jarsky et al., 2008; Kim and Spruston, 2012). Most notably, we observed that the input resistance was substantially higher in RS neurons compared to BS neurons. Consistent with this finding, the rheobase was lower and the discharge frequency in response to depolarizing pulses was higher in RS neurons, indicating a higher intrinsic excitability of this type of PC. A difference in input resistance was previously observed by Menendez de la Prida et al. (2003), albeit only when comparing BS neurons to a subset of RS neurons displaying adaptation. Jarsky et al. (2008) also reported similar differences along the proximal-distal axis of the subiculum, corresponding to the differential distribution of BS and RS neurons (Kim and Spruston, 2012; Cembrowski et al., 2018). In our study, we further found a larger sag potential in response to hyperpolarizing pulses in RS compared to BS neurons. Similar observations were reported by Kim and Spruston (2012), whereas Menendez de la Prida et al. (2003) found no sag response in RS neurons, probably due to the smaller hyperpolarizing pulses tested. Finally, we detected differences in the AP waveform and its afterpotentials. The rise rate of the AP was lower, and the fast and medium AHP were substantially smaller in BS neurons compared to RS neurons (Behr et al., 1996; Staff et al., 2000; Kim and Spruston, 2012). These differences further underly the functional differentiation of the two PC types in the subiculum.

### 4.2 Perisomatic inhibition is stronger in RS compared to BS PCs

When comparing the evoked monosynaptic IPSPs at minimal stimulus intensities in the cell body layer, we found that RS cells consistently receive larger amplitude IPSPs than BS neurons. This result is in contrast to the observation of De la Prida (2003), that BS neurons show a stronger inhibitory synaptic input when compared to RS neurons. However, this difference might be due to the fact that de la Prida et al. stimulated the alveolus, antidromically activating feedback inhibition, plausibly involving a diverse set of interneurons. In our study, we used local orthodromic stimulation in the cell body layer to recruit pharmacologically isolated, monosynaptic perisomatic inhibition.

Larger IPSP amplitudes in RS neurons are primarily caused by a higher input resistance of this PC type, as indicated by the comparable IPSC amplitudes in voltage-clamp recordings. However, it is also feasible that RS and BS PCs receive different levels of perisomatic inhibitory input. In fact, our immunohistochemical labeling for PV, the major marker for perisomatic inhibitory interneurons (Booker and Vida, 2018), suggests that the superficial cell body layer, where most of the recorded RS neurons were localized, contains a higher density of PV axon collaterals (see Figure 1C) than the deeper layers, where the majority of BS neurons was recorded. A similar gradient of PV interneuron-mediated inhibition was previously described in the CA1 area (Lee et al., 2014).

In addition to the stronger effective inhibition, as reflected by the larger IPSPS, RS neurons also exhibited a stronger paired pulse depression, indicating differences to BS neurons in short term plasticity at the perisomatic input. However, voltage-clamp recordings showed that the kinetics of IPSCs, as well as the pharmacological sensitivity of the evoked response to presynaptic modulation by an M2R agonist are comparable and consistent with PV IN-mediated inhibition in both PC types (Booker et al., 2017).

### 4.3 Cell-type specific iLTP in RS and BS PCs

Our findings demonstrate, that plasticity can be induced at perisomatic inhibitory synapses onto subicular PCs, but shows cell type-specific differences in its expression. These findings complement earlier observations that excitatory synaptic plasticity also shows PC type-dependent differences in this region (Wozny et al., 2008b). While iLTP was readily induced in both PC types when an associative stimulus, involving simultaneously pre-and postsynaptic activation, was applied, a non-associative, presynaptic stimulation paradigm induced iLTP solely in RS neurons. Despite this difference, in both cell types, iLTP showed a strong dependence on postsynaptic calcium signaling and a postsynaptic expression is further indicated by the unchanged PPR and 1/CV^2^ before and after expression of iLTP.

Our findings provide the first evidence for iLTP expression in the subiculum. Our data thus adds to the growing diversity of inhibitory plasticity observed in the hippocampus (Patenaude et al., 2003; Jappy et al., 2016) and other cortical areas (Maffei, 2011; Galanis and Vlachos, 2020; Capogna et al., 2021; Chapman et al., 2022). In the adjacent CA1 region, Patenaude et al. (2003) showed that theta burst-patterned stimulation (TBS), but not HFS, induced iLTP in CA1 PCs. This iLTP relied on the activation of group I/II mGluRs and postsynaptic calcium. While we have not tested for the involvement of different receptors, G-protein coupled downward signaling, either via GABA-B receptors (Schiller et al., 1998; Higley, 2014; Gandolfi et al., 2020) or metabotropic glutamate receptors (Patenaude et al., 2003; Yoshioka et al., 2010; Bannai et al., 2015) are likely candidates as molecular mechanism.

### 4.4 Functional implications

Previous studies suggest that subicular BS and RS PCs play distinct roles within the subicular network (De La Prida, 2003; Wozny et al., 2008a,b; Behr et al., 2009; Kim and Spruston, 2012; Böhm et al., 2015; Cembrowski et al., 2018). In terms of network connectivity, it has been shown that the subiculum exhibits an asymmetrical internal wiring scheme (Böhm et al., 2015): Both cell types show monosynaptic recurrent excitatory connections, however, only RS neurons form excitatory synapses onto BS neurons, BS do not provide synapses onto RS neurons. The two types, thus, appear to constitute two subnetworks with a unidirectionally coupling between them. Considering the higher excitability of RS neurons, combined with a stronger and more plastic inhibitory input, as compared to BS neurons, the following picture emerges: At low levels of synaptic input from the CA1, RS neurons will be recruited first and process information under a tight inhibitory control. At increasing excitation levels, BS neurons will also be recruited, promoted additionally by an overflow of excitation from the RS to the BS subnetwork via the unidirectional recurrent connections. Under these conditions, the weaker inhibition onto BS neurons may ultimately enable a non-linear amplification within this subnetwork. Given the differential output connectivity of RS and BS neurons (Kim and Spruston, 2012; Cembrowski et al., 2018), the input intensity-dependent dynamic recruitment of the RS vs. BS subnetworks would translate into a progressive shift in the channeling of information to cortical and subcortical target areas. In this functional context, inhibitory plasticity is instrumental to maintain the divergent balance of excitation and inhibition onto the two cell types, supporting their division of labor in information processing.

## Data availability statement

The raw data supporting the conclusions of this article will be made available by the authors, without undue reservation.

## Ethics statement

The animal study was approved by LaGeSo Berlin, Germany (Nr.: T-CH 0020/40). The study was conducted in accordance with the local legislation and institutional requirements.

## Author contributions

AG: Writing – review & editing, Writing – original draft, Visualization, Validation, Project administration, Investigation, Formal Analysis, Data curation, Conceptualization. SG: Writing – review & editing, Validation, Supervision, Conceptualization. JB: Writing – review & editing, Validation, Supervision, Resources, Funding acquisition, Conceptualization. IV: Writing – review & editing, Writing – original draft, Validation, Supervision, Resources, Funding acquisition, Data curation, Conceptualization. DÖ: Investigation, Writing – review & editing, Writing – original draft.

## Footnotes

^1^http://www.stimfit.org

^2^http://fiji.sc/wiki/index/php/FIJI

^3^https://neutracing.com

^4^https://www.graphpad.com

## Supplementary material

SUPPLEMENTARY FIGURE S1 IPSCs underlying the evoked synaptic responses in BS and RS cells. **(A)** Representative traces illustrate evoked IPSCs and IPSPs in voltage clamp (VC) and current clamp (CC) recordings from a BS (top, in blue) and a RS (bottom, in red) PC. **(B)** Summary plots of IPSC peak amplitudes (top left), onset latencies (top right), 20 - 80% rise times (bottom left) and decay time constants (bottom right). Each dot represents averaged results over a 5-min period for individual cells (BS = 8; RS = 9) and data is represented as mean ± SEM.
