# Supplementary figures and images for: Cell-type specific inhibitory plasticity in subicular pyramidal cells
Source: Front Cell Neurosci. 2024 Apr 23;18:1368627. doi: 10.3389/fncel.2024.1368627 (PMC11074406; doi:10.3389/fncel.2024.1368627)

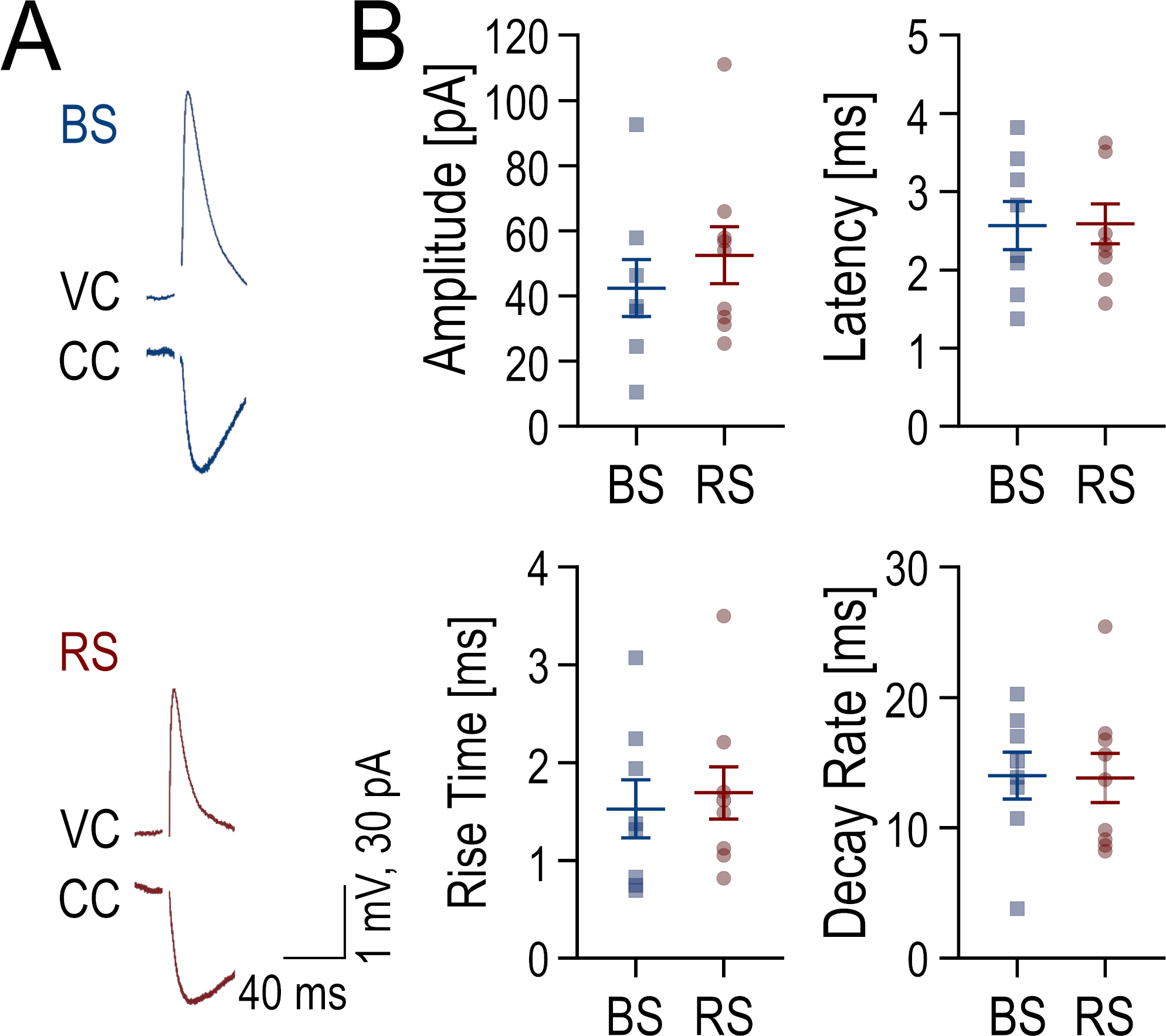

Supplement: SUPPLEMENTARY FIGURE S1 — IPSCs underlying the evoked synaptic responses in BS and RS cells. (A) Representative traces illustrate evoked IPSCs and IPSPs in voltage clamp (VC) and current clamp (CC) recordings from a BS (top, in blue) and a RS (bottom, in red) PC. (B) Summary plots of IPSC peak amplitudes (top left), onset latencies (top right), 20 - 80% rise times (bottom left) and decay time constants (bottom right). Each dot represents averaged results over a 5-min period for individual cells (BS = 8; RS = 9) and data is represented as mean ± SEM. [file Image_1.TIFF]
